# Lipid and High-Density Lipoprotein (HDL) Subfraction Changes in Premenopausal and Postmenopausal Women With Breast Cancer Receiving Hormonal Therapy

**DOI:** 10.7759/cureus.108701

**Published:** 2026-05-12

**Authors:** Anastasia Nikolakopoulou, Konstantinos Nastos, Dimitrios Papakonstantinou, Kalliopi E Stavrati, Vasiliki Michalaki, Georgios Koutroulis, Nikolaos Dafnios, Ioannis Papakonstantinou, Christos Papadimitriou

**Affiliations:** 1 Second Department of Surgery, Aretaieion University Hospital, National and Kapodistrian University of Athens, Athens, GRC; 2 Third Department of Surgery, Attikon University Hospital, National and Kapodistrian University of Athens, Athens, GRC; 3 Department of Surgery, Evgenideion Hospital, National and Kapodistrian University of Athens, Athens, GRC; 4 Department of Oncology, Aretaieion University Hospital, National and Kapodistrian University of Athens, Athens, GRC; 5 Department of Cardiology, Hygeias Melathron, TYPET, Athens, GRC

**Keywords:** aromatase inhibitors, breast cancer, endocrine therapy, hdl subfractions, lipid profile, tamoxifen

## Abstract

Background and aim: Hormonal modulation therapy is an important component of hormone receptor-positive breast cancer treatment. However, the type and duration of hormonal therapies have distinct effects on lipid profile parameters and cardiovascular disease risk. This study aimed to investigate the impact of tamoxifen and aromatase inhibitors (AIs) on lipid profiles and high-density lipoprotein (HDL) subfractions in women with breast cancer.

Materials and methods: In this retrospective observational study, 100 women were included (50 healthy controls and 50 patients with histologically confirmed breast cancer). Among breast cancer patients, 25 were premenopausal receiving tamoxifen treatment, and 25 were postmenopausal receiving AI therapy, based on routine clinical practice. Lipid parameters were assessed at baseline and following six months of treatment in all participants. Premenopausal patients received tamoxifen, while postmenopausal patients were treated with AIs. Serum total cholesterol, triglycerides, low-density lipoprotein cholesterol (LDL-C), HDL cholesterol, and HDL subfractions (HDL-2 and HDL-3) were analyzed.

Results: In the tamoxifen-treated premenopausal cohort, total cholesterol decreased by 8.48% and LDL-C by 15.94%, with redistribution toward larger HDL particles, reflected by an increase in HDL-2 of 21.1% and a decrease in HDL-3 of 12.06% (all p<0.001). In the postmenopausal aromatase inhibitor cohort, a mixed lipid pattern was observed, with increases in total cholesterol (+3.38%) and LDL-C (+7.02%), decreases in HDL-C (-1.56%) and HDL-2 (-20.75%), an increase in HDL-3 (+8.69%), and a decrease in triglycerides (-10.95%) (all p<0.001). Direct comparison of change scores between cohorts showed significant differences for all examined lipid parameters (all p<0.001).

Conclusion: Distinct six-month trajectories in lipid profile parameters and HDL subfractions were observed between premenopausal women receiving tamoxifen and postmenopausal women receiving aromatase inhibitors. These findings likely reflect the combined influence of endocrine therapy and underlying menopausal physiology and should not be interpreted as a direct causal head-to-head comparison of isolated drug effects.

## Introduction

Breast cancer is the most commonly diagnosed cancer and a leading cause of cancer-related mortality among women worldwide [1]. Advances in early detection and treatment have substantially improved long-term survival, resulting in a growing population of survivors at increased risk of non-cancer morbidity, particularly cardiovascular disease, which contributes to late mortality through treatment-related metabolic effects and shared risk factors such as age and lifestyle [2].

Lipid abnormalities represent one plausible pathway through which systemic therapy may affect long-term cardiovascular risk. In addition to conventional lipid parameters, such as total cholesterol (TC) and low-density lipoprotein cholesterol (LDL-C), high-density lipoprotein cholesterol (HDL-C) subfractions may provide a more nuanced view of lipid remodeling during endocrine therapy [3].

Existing literature has reported heterogeneous findings regarding the lipid effects of endocrine therapy. Tamoxifen has generally been associated with reductions in TC and LDL-C [3,4], whereas aromatase inhibitor-related findings appear more variable, potentially depending on the specific agent, study design, treatment duration, and prior endocrine exposure [5]. Moreover, menopause itself is associated with adverse changes in lipid profile and cardiovascular risk, which complicates the interpretation of non-randomized treatment comparisons [6].

Beyond conventional lipid measures, lipoprotein subfractions may offer deeper insight into cardiovascular risk. In untreated breast cancer patients, alterations in HDL subfractions have been observed, including decreased HDL-2 and elevated atherogenic ratios, indicating an unfavorable lipid profile that may predispose to cardiovascular disease [7].

The present study aimed to describe changes in conventional lipid parameters and HDL subfractions over six months in the following two clinically distinct endocrine therapy cohorts: premenopausal women treated with tamoxifen and postmenopausal women treated with aromatase inhibitors. We hypothesized that these two routine-care hormonal therapy pathways would be associated with different patterns of lipid remodeling, with a more favorable profile expected in the tamoxifen-treated cohort.

## Materials and methods

Study design

A retrospective observational study was conducted on consecutive female patients with hormone receptor-positive breast cancer managed at the Oncology Unit of the Aretaieion University Hospital between April 2019 and April 2024. The patient cohort was comprised of 50 healthy controls from the community and 50 women with histologically confirmed breast cancer. Among patients with breast cancer, 25 were premenopausal and had paired baseline and six-month measurements after initiation of tamoxifen, while 25 were postmenopausal and had paired baseline and six-month measurements after initiation of aromatase inhibitor therapy. Healthy controls were stratified by menopausal status into 25 premenopausal and 25 postmenopausal women. The study was conducted in accordance with the Declaration of Helsinki.

Inclusion and exclusion criteria

Female patients with histologically confirmed hormone receptor-positive breast cancer and available baseline lipid measurements prior to initiation of systemic therapy were included in the study. Patients were excluded from the study if they had conditions known to affect lipid metabolism, including diabetes mellitus, thyroid disease, nephrotic syndrome, acute infection, chronic inflammatory disease, or cancer-related cachexia. Patients receiving lipid-lowering medication, such as statins, were also excluded.

Treatment allocation

Hormonal therapy was administered according to standard clinical practice [8]. Premenopausal patients received tamoxifen, whereas postmenopausal patients were treated with aromatase inhibitors, including anastrozole, letrozole, or exemestane. The choice of endocrine agent was based on menopausal status and clinical indication and was independent of the present study.

Data collection and laboratory analysis

Venous blood samples were obtained after a 12-h overnight fast. A total of 7 mL of venous blood was collected from each participant, centrifuged at 1000 g for 10 min, and serum samples were stored at -70°C until analysis. Serum lipid parameters measured included total cholesterol, triglycerides, low-density lipoprotein cholesterol, high-density lipoprotein cholesterol, and the HDL subfractions HDL-2 and HDL-3, which were measured using density gradient ultracentrifugation. All lipid measurements were expressed in mg/dL and performed using standard laboratory methods routinely employed in clinical lipid analysis [9]. For patients receiving endocrine therapy, lipid measurements were performed at baseline, prior to initiation of treatment, and after six months of continuous therapy.

Statistical analysis

Continuous variables are presented as mean±standard deviation. Baseline comparisons between controls and patients were performed separately for premenopausal and postmenopausal groups using one-way analysis of variance (ANOVA). Within-cohort changes from baseline to six months were evaluated using paired t-tests. For direct comparison of longitudinal changes between cohorts, individual change scores were calculated as six months minus baseline and compared using Welch’s t-test. Results are reported with effect estimates, 95% confidence intervals, and p-values. Because treatment assignment was structurally linked to menopausal status, between-cohort comparisons were interpreted as exploratory and descriptive rather than as estimates of isolated pharmacologic effect. Due to the observational nature of the study, no formal power analysis was undertaken. All tests were two-sided, and a p<0.05 was considered statistically significant. Statistical analyses were performed using SPSS software version 27 (Chicago, IL: IBM Corp.).

## Results

The total study population consisted of 50 healthy controls and 50 women with breast cancer with paired baseline and six-month measurements after initiation of endocrine therapy. Controls and patients were analyzed separately by menopausal status, which also dictated hormonal treatment (tamoxifen for premenopausal patients and aromatase inhibitors {AIs} for postmenopausal patients). No statistically significant differences in age and baseline lipid values were observed between control and breast cancer patients (Tables 1, 2).

**Table 1:** Baseline demographic and lipid characteristics of premenopausal healthy controls and breast cancer patients. Lipid parameters were measured prior to initiation of any systemic therapy. Comparisons were performed using ANOVA. Values are presented as mean±standard deviation. LDL-C: low-density lipoprotein cholesterol; HDL-C: high-density lipoprotein cholesterol

Parameters	Control (n=25)	Breast cancer (n=25)	F-statistic	p-Value
Age (years)	40.56±3.58	41.58±3.65	1.38	0.24
Total cholesterol (mg/dL)	183.84±14.24	184.84±14.24	0.09	0.77
HDL-C (mg/dL)	62.92±6.16	60.92±6.16	1.80	0.18
HDL-2 (mg/dL)	25.72±2.37	24.96±2.39	1.63	0.21
HDL-3 (mg/dL)	37.20±3.86	35.79±3.78	2.19	0.14
LDL-C (mg/dL)	100.68±16.95	104.08±16.86	0.74	0.39
Triglycerides (mg/dL)	100.84±16.13	98.84±16.13	0.32	0.57

**Table 2:** Baseline demographic and lipid characteristics of postmenopausal healthy controls and breast cancer patients. Lipid parameters were measured prior to initiation of any systemic therapy. Comparisons were performed using ANOVA. Values are presented as mean±standard deviation. LDL-C: low-density lipoprotein cholesterol; HDL-C: high-density lipoprotein cholesterol

Parameters	Control (n=25)	Breast cancer (n=25)	F-statistic	p-Value
Age (years)	68.56±3.58	69.56±3.69	0.37	0.52
Total cholesterol (mg/dL)	213.56±14.40	215.56±14.40	0.34	0.56
HDL-C (mg/dL)	63.44±6.83	62.44±6.83	0.36	0.55
HDL-2 (mg/dL)	22.20±2.36	21.84±2.30	0.40	0.53
HDL-3 (mg/dL)	41.24±4.49	40.60±4.55	0.32	0.57
LDL-C (mg/dL)	127.04±16.53	129.56±16.64	0.38	0.54
Triglycerides (mg/dL)	115.16±23.38	118.16±23.38	0.25	0.62

Premenopausal patients

Among premenopausal patients treated with tamoxifen, significant changes in lipid parameters were observed after six months of therapy (Table 3). Total cholesterol (TC) decreased significantly from 186.84±14.24 mg/dL at baseline to 171±11.55 mg/dL after treatment (p<0.001), accompanied by a marked reduction in LDL-C from 107.88±16.89 mg/dL to 90.68±14.10 mg/dL (p<0.001). Total HDL-C increased modestly from 58.92±6.16 mg/dL to 59.80±6.27 mg/dL (p<0.001). More importantly, HDL subfractions shifted toward a potentially more favorable profile, with HDL-2 increasing from 24.08±2.45 mg/dL to 29.16±3.12 mg/dL (p<0.001) and HDL-3 decreasing from 34.84±3.74 mg/dL to 30.64±3.17 mg/dL (p<0.001). Triglycerides increased slightly from 99.84±16.13 mg/dL to 102.96±16.60 mg/dL (p<0.001).

**Table 3:** Changes in lipid parameters after six months of tamoxifen therapy in premenopausal patients. Values are presented as mean±standard deviation. P-values refer to within-group comparisons (paired t-test). LDL-C: low-density lipoprotein cholesterol; HDL-C: high-density lipoprotein cholesterol

Parameters	Baseline (mg/dL)	6 months (mg/dL)	t-Value	p-Value
Total cholesterol	186.84±14.24	171±11.55	-26.4	<0.001
HDL-C	58.92±6.16	59.8±6.27	13.33	<0.001
HDL-2	24.08±2.45	29.16±3.12	33.42	<0.001
HDL-3	34.84±3.74	30.64±3.17	-29.58	<0.001
LDL-C	107.88±16.89	90.68±14.10	-30.6	<0.001
Triglycerides	99.84±16.13	102.96±16.60	29.43	<0.001

Postmenopausal patients

Among postmenopausal women treated with aromatase inhibitors, total cholesterol increased from 216.56±14.40 mg/dL to 223.88±15 mg/dL (p<0.001), and LDL-C increased from 131.04±16.53 mg/dL to 140.24±17.62 mg/dL (p<0.001) (Table 4). HDL-C decreased modestly from 61.44±6.83 mg/dL to 60.48±6.76 mg/dL (p<0.001). HDL subfraction remodeling was directionally unfavorable, with HDL-2 decreasing from 21.4±2.47 mg/dL to 16.96±1.84 mg/dL (p<0.001) and HDL-3 increasing from 40.04±4.38 mg/dL to 43.52±4.96 mg/dL (p<0.001). In contrast to the worsening pattern in total cholesterol, LDL-C, and HDL subfractions, triglycerides decreased from 120.16±23.38 mg/dL to 107.00±25.08 mg/dL (p<0.001).

**Table 4:** Changes in lipid parameters after six months of aromatase inhibitor therapy in postmenopausal patients. Values are presented as mean±standard deviation. P-values refer to within-group comparisons (paired t-test). LDL-C: low-density lipoprotein cholesterol; HDL-C: high-density lipoprotein cholesterol

Parameters	Baseline (mg/dL)	6 months (mg/dL)	t-Value	p-Value
Total cholesterol	216.56±14.39	223.88±15	32.97	<0.001
HDL-C	61.44±6.82	60.48±6.76	-7.06	<0.001
HDL-2	21.4±2.46	19.96±1.84	-31.27	<0.001
HDL-3	40.04±4.38	43.52±4.95	18.91	<0.001
LDL-C	131.04±16.53	140.24±17.62	41.07	<0.001
Triglycerides	120.16±23.38	107.00±25.08	-17.41	<0.001

Comparative lipid profiles

Direct comparison of change scores between the tamoxifen-treated premenopausal cohort and the aromatase inhibitor-treated postmenopausal cohort showed significant differences for total cholesterol, LDL-C, HDL-C, HDL-2, HDL-3, and triglycerides (all p<0.001; Table 5). The tamoxifen-treated premenopausal cohort showed reductions in total cholesterol and LDL-C, together with a redistribution toward larger HDL subfractions, reflected by increased HDL-2 and decreased HDL-3 cholesterol. In contrast, the aromatase inhibitor-treated postmenopausal cohort showed a mixed conventional lipid pattern, with increases in total cholesterol and LDL-C and a decrease in triglycerides, together with an opposite pattern of HDL subfraction remodeling characterized by decreased HDL-2 and increased HDL-3 cholesterol. These between-cohort differences suggest distinct lipid trajectories during the two endocrine-treatment pathways; however, they should be interpreted descriptively because treatment exposure was determined by menopausal status rather than by random allocation.

**Table 5:** Comparison of treatment-related changes in lipid parameters (six months vs. baseline) between tamoxifen-treated and aromatase inhibitor-treated patients. Values are presented as mean±standard deviation. Between-group comparisons were performed using independent-samples t-tests with Welch’s correction. The symbol “Δ” denotes the change in lipid parameter values between baseline and the 6-month follow-up measurement (6-month value minus baseline value). LDL-C: low-density lipoprotein cholesterol; HDL-C: high-density lipoprotein cholesterol

Parameters	Δ Tamoxifen (mg/dL)	Δ Aromatase inhibitors (mg/dL)	t-Value	p-Value
Total cholesterol	-15.84±3	+7.32±1.11	-36.26	<0.001
HDL-C	+0.88±0.33	-0.96±0.68	12.22	<0.001
HDL-2	+5.08±0.76	-4.44±0.71	45.73	<0.001
HDL-3	-4.2±0.71	+3.48±0.92	-33.13	<0.001
LDL-C	-17.2±2.81	+9.2±1.12	-43.6	<0.001
Triglycerides	+3.12±0.53	-13.16±3.78	21.32	<0.001

## Discussion

In this retrospective cohort study, the two endocrine treatment pathways were associated with distinct lipid trajectories over six months. The tamoxifen-treated premenopausal cohort showed reductions in total cholesterol (-8.48%) and LDL-C (-15.94%), a slight increase in HDL-C (+1.49%), an increase in HDL-2 (+21.10%), a decrease in HDL-3 (-12.06%), and a small increase in triglycerides (+3.12%). In contrast, the aromatase inhibitor-treated postmenopausal cohort showed a mixed conventional lipid pattern, with increases in total cholesterol (+3.38%) and LDL-C (+7.02%), a slight decrease in HDL-C (-1.56%), a marked decrease in HDL-2 (-20.75%), an increase in HDL-3 (+8.69%), and a decrease in triglycerides (-10.95%). These findings should be interpreted as descriptive differences between two clinically distinct cohorts rather than as a direct causal comparison of isolated drug effects. Because endocrine therapy in this cohort was determined by menopausal status rather than random allocation, the observed differences likely reflect the combined influence of endocrine therapy and underlying menopausal physiology.

The favorable effects of tamoxifen on TC and LDL-C observed in this study are consistent with previous reports in both randomized and observational settings. Tamoxifen has long been recognized to produce estrogen-like effects in hepatic tissue, leading to reduced hepatic cholesterol synthesis and increased LDL receptor expression, thereby promoting LDL clearance from the circulation [3]. Meta-analyses of randomized trials have confirmed reductions in total cholesterol and LDL cholesterol during tamoxifen therapy, although effects on HDL cholesterol and triglycerides have been less consistent across studies [4]. The present findings extend this literature by demonstrating that tamoxifen not only modifies conventional lipid parameters but also induces a marked redistribution of HDL subfractions, favoring HDL-2 over HDL-3.

In contrast, the lipid effects of aromatase inhibitors have been more heterogeneous in the literature. Several studies have reported neutral or adverse changes in lipid profiles during AI therapy, including reductions in HDL cholesterol and increases in LDL cholesterol or total cholesterol, although findings have varied depending on study design, follow-up duration, and patient population [2]. The estrogen deprivation induced by AIs represents a plausible explanation for these observations, as estrogen plays a pivotal role in regulating hepatic lipid metabolism, lipoprotein lipase activity, and HDL particle composition [10]. In the present analysis, AI therapy was associated with a shift toward smaller HDL particles, reflected by reduced HDL-2 and increased HDL-3 cholesterol, a pattern that contrasts sharply with that observed during tamoxifen treatment.

HDL is increasingly recognized as a heterogeneous lipoprotein class, and its cardioprotective properties may depend more on particle composition and functionality than on total HDL cholesterol concentration alone. Larger HDL-2 particles are generally considered more efficient mediators of reverse cholesterol transport, whereas smaller HDL-3 particles may reflect altered HDL remodeling and reduced atheroprotective capacity [11,12]. Epidemiologic studies in non-cancer populations have demonstrated associations between low HDL-2 levels or low HDL-2/HDL-3 ratios and increased cardiovascular risk, particularly in women [5]. In breast cancer patients, alterations in HDL subfractions have been reported even prior to treatment, suggesting that the disease itself may be associated with an unfavorable lipid phenotype [7,13].

The opposing lipid remodeling observed between tamoxifen and aromatase inhibitor therapy likely reflects differences in their molecular mechanisms of action. Tamoxifen acts as a selective estrogen receptor modulator, exerting effects in the liver that influence apolipoprotein expression, lipoprotein assembly, and cholesterol transport pathways. These effects may be mediated by molecular pathways that involve both dependent and independent estrogen receptor signaling [14,15]. In contrast, aromatase inhibitors suppress systemic estrogen synthesis, leading to estrogen deprivation that may adversely affect hepatic lipid regulation [16]. Mechanistically, this may reflect differences in hepatic lipid handling associated with selective estrogen receptor modulation and estrogen deprivation, although the present study design does not allow these pathways to be disentangled from menopause-related physiology.

From a clinical perspective, these results should not be interpreted as evidence of cardiovascular benefit or harm, because cardiovascular outcomes were not measured. Rather, they suggest that the two endocrine-treatment pathways commonly encountered in routine practice may be associated with different biomarker trajectories, supporting individualized lipid surveillance and cardiovascular risk assessment during endocrine therapy and survivorship. A major limitation of this study is confounding by indication, because endocrine therapy was selected according to menopausal status in routine practice, and menopause is an important determinant of lipid profile and cardiovascular risk. Additional limitations include the retrospective observational design, the modest sample size, the absence of clinical cardiovascular endpoints, and the possibility of residual confounding by unmeasured factors.

## Conclusions

In conclusion, this study demonstrates that premenopausal women treated with tamoxifen and postmenopausal women treated with aromatase inhibitors exhibit distinct six-month trajectories of conventional lipid parameters and HDL subfractions. The tamoxifen-treated cohort was associated with lower total cholesterol and LDL-C, together with a shift toward larger HDL subfractions, whereas the aromatase inhibitor-treated cohort showed a mixed conventional lipid profile accompanied by an opposite HDL subfraction pattern. These findings likely reflect the combined influence of endocrine therapy and menopausal physiology and support context-specific lipid monitoring and cardiovascular risk assessment during breast cancer survivorship. However, they should not be interpreted as a direct, head-to-head causal comparison of isolated drug effects.
